# Silicon Mechanisms to Ameliorate Heavy Metal Stress in Plants

**DOI:** 10.1155/2018/8492898

**Published:** 2018-04-22

**Authors:** Abolghassem Emamverdian, Yulong Ding, Yinfeng Xie, Sirous Sangari

**Affiliations:** ^1^Co-Innovation Center for Sustainable Forestry in Southern China, Nanjing Forestry University, Nanjing 210037, China; ^2^College of Biology and the Environment, Nanjing Forestry University, Nanjing 210037, China; ^3^Bamboo Research Institute, Nanjing Forestry University, Nanjing 210037, China; ^4^College of Chemical Engineering, Nanjing Forestry University, Nanjing 210037, China

## Abstract

The increased contaminants caused by anthropogenic activities in the environment and the importance of finding pathways to reduce pollution caused the silicon application to be considered an important detoxification agent. Silicon, as a beneficial element, plays an important role in amelioration of abiotic stress, such as an extreme dose of heavy metal in plants. There are several mechanisms involved in silicon mediation in plants, including the reduction of heavy metal uptake by plants, changing pH value, formation of Si heavy metals, and stimulation of enzyme activity, which can work by chemical and physical pathways. The aim of this paper is to investigate the major silicon-related mechanisms that reduce the toxicity of heavy metals in plants and then to assess the role of silicon in increasing the antioxidant enzyme and nonenzyme activities to protect the plant cell.

## 1. Introduction

Silicon is a beneficial mineral element commonly found in soil. It is the second most abundant element after oxygen [1, 2] in soil. Since silicon is an essential element and a plant nutrient [3, 4], it has an important role in plant growth, yield [3, 5, 6], photosynthetic properties, chlorophyll contents, and enzyme activity [7–9], especially under stressful conditions [1, 10]. Currently, there is no evidence showing the direct role of silicon in plant metabolism. However, silicon can still be assumed to be essential in the process because it belongs to a molecular compound which is involved in plant metabolism. Because plants require silicon, it helps with plant growth and development. Consequently, silicon reduction can adversely affect plant growth and produce abnormal characters in plants [11, 12]. Many researchers confirmed that silicon has the ability to ameliorate the abiotic stresses such as an excess dose of heavy metal and increase the tolerance of plants against heavy metals [7, 13–15]. The mechanism by which silicon alleviates stress from heavy metals can be categorized into internal and external mechanisms [16, 17]. In the external mechanism, silicon ameliorates the heavy metals' toxicity through various methods, such as reducing the absorption and activity of the metal or changing the metal's formation by adding a silicon compound. However, in the internal mechanisms, silicon reduces the adverse effects of heavy metal toxicity through different mechanisms, such as stimulation of antioxidant enzyme activity, complexation and compartmentalization of silicon with metal ions, and changing the cell wall by transportation control [17]. In addition, silicon can protect plants exposed to biotic and abiotic stresses [18, 19]. The protective role of silicon revealed in plants can be attributed to an accumulation of polysialic acid in plant cells. Thus, with an enhancement in polysialic acid concentration in the cell wall, plant tolerance increases and indirectly interferes with stress factors [20]. The application of silicon on tissue cultures can protect the cell wall by decreasing oxidative stress, ameliorating the plant growth cycles, including embryogenesis, organogenesis, and inducing growth in traits and leaves in vitro [21]. Moreover, the protective role of silicon in reactive oxygen species (ROS) scavenging is bold. Silicon can scavenge ROS indirectly. A previously conducted experiment shows that Si can decrease OH hydroxyl radical accumulation in cucumber leaves by reducing the free apoplastic Mn^+2^ [19]. One of the other protective roles of silicon is regulating internal water in plants [22] so that the accumulation of silicon in epidermal tissue preserves water in the transpiration process [23]. Generally, plants tend to accumulate heavy metals, such as cadmium, in the root more than shoot and stem [24]. Therefore, silicon is accumulated in plants' roots for certain mechanisms, including a physical barrier, reduction of translation, and reduction of heavy metal uptake.

## 2. Silicon Defense Mechanisms

Silicon is often absorbed in plants through monosilicic acid formation and precipitated in internal plant parts, such as cell wall and lumens. In addition, it is deposited as an amorphous silica (Opal A; Sio_2_·*n*H_2_o) or in intercellular sites like phytoliths [25-10]. Phytolith is a Greek word meaning “stone of plant,” which is known to be the space between the plant cells [25]. Silicon's defense mechanisms appear throughout plants. In leaves silicon is used to create structures such as epidermal trichomes and hair. Silicon is also accumulated in spines as amorphous silica (SiO_2_) and phytoliths [26]. In plants and soil, there are different mechanisms and pathways by which silicon scavenges ROS and ameliorates heavy metals. In growth media (i.e., tissue culture), silicon decreases the ions' activities and limits the metal uptake and metal translocation from roots to shoots. In cell structure, silicon ameliorates heavy metal stress through various mechanisms, such as regulating the gene expression involved in metal transport and metal-chelation mechanisms, participating in coprecipitation of metals, changing the plant structure, and stimulating antioxidant enzymes' activity [13, 18, 27]. Song et al. indicated that, with the combination of silicon and cadmium, cadmium tolerance increases in* B. chinensis* demonstrating the role of silicon in the reduction of heavy metal uptake, limitation of root to shoot translocation, and stimulation of antioxidant enzyme activity [28]. Many researchers reported that codeposit of metal with silicon can reduce the concentration of toxic ions in plants [13, 17, 29]. It has been reported that, using some mechanisms, such as root exudation and pH increase, silicon limits the aluminum uptake in roots; this issue can later precipitate Al concentration in the root surface [30, 31]. In a general classification, silicon detoxification mechanisms can be grouped as either chemical or physical mechanisms. Chemical mechanisms refer to the mechanisms involved in coprecipitation of heavy metals with silicon, while in physical mechanisms silicon reduces the translocation of heavy metals to shoot and aerial parts by changing the plant structure such as the apoplastic barrier [32]. Generally, Si is involved in the alleviation process in plants exposed to abiotic stresses and heavy metals in some important mechanisms, including (1) stimulation of antioxidant enzyme activity to enhance ROS scavenging, (2) complexation and immobilization of toxic metal ions in plants [18, 33], (3) deposition and accumulation in plant tissue for developing the rigidity and stability in leaves, (4) water mobility, and (5) providing plant nutrient and coprecipitation of metal toxicity [34]. In the following, we discuss some of the key mechanisms involved in silicon detoxification in plants.

### 2.1. Mechanism One: Reduction of Heavy Metal Uptake by Plants

Regarding the relationship between the silicon and heavy metal uptake, it can be stated that silicon can alleviate and reduce the uptake of heavy metal and its transportation in plants [13, 28, 35, 36]. Moreover, silicon can increase the chelated ions through (1) stimulation of root exudate, which can limit metal uptake by roots in plants [13] or (2) decreasing the free metals in plant organs which reduces the translocation activity in apoplasm [13]. In the cell wall, silicon can accumulate in the lignin and improve the metal binding; it can then reduce the translocation of ions from roots to aerial organs [37, 38]. Silicon creates a complex with metal ions in the cell wall and additionally precipitates metal ions as a cofactor [30]. The results revealed that enhancement of silicon could increase malic and formic acid in plants growing process and consequently reduce the uptake of Al [30]. Furthermore, it was observed that phenolic compounds in maize can decrease the Al uptake so that flavonoid-phenolics can lead to Al-chelating link and reduce the Al uptake in plants [13]. Heavy metal chelating significantly contributes to digestion of heavy metals, and it is created by chelation of heavy metal with flavonic-phenolics or other organic acids [39]. Silicon can increase heavy metal accumulation in intercellular plant parts [13]. By investigating the amelioration role of silicon in Mn, Doncheva indicated that silicon, used as a barrier, can expand the epidermal layer of maize. It can lead to an accumulation of Mn in nonphotosynthetic tissue [40]. Then, through certain mechanisms, such as coprecipitation, it prevents the heavy metals from translocating to other plant parts. It is reported by Hai-Hong Gu that the reduction in stem-to-shoot translocation in rice was the consequence of a high concentration of silicon [32].

Most beneficial effects of silicon can be revealed in the accumulation in the cell walls of root, stems, leaves, and hulls, which enhance the stability of plant tissue as a physical barrier [37]. In roots, silicon increases the binding of metal ions by decreasing the apoplastic bypass flow and reduces the translocation of toxic metals from roots to shoots. Moreover, accumulation of Si in the cell wall of stems, leaves, and hulls limits the transpiration of the cuticle with alternation in the efficiency and function of the cell wall and consequently increases the plant's resistance against stresses [41]. Rizwan et al. reported that silicon decreases cadmium uptake and reduces the translocation of cadmium to shoots [42]. In the case of silicon precipitation in shoots, silica precipitates in water evaporation sites of the plant's shoot as phytoliths. This can be close to the epidermis of the plant shoot [42–44]. Additionally, silicon can shift the Mn to the leaf blade causing a homogenetic mechanism against Mn and then decrease Mn uptake [45]. Thus, it can be estimated that beneficial impacts on plants can be obtained by high deposition in shoots [46]. However, most deposition in plants depends on Si uptake by roots [37]; therefore, plants with less ability to uptake silicon could be deprived of silicon benefits [45]. The roots are the first line exposed to heavy metals; this issue shows the role of root anatomy in reducing heavy metal toxicity. Apoplastic barriers in roots, including exodermis, epiblema, and endodermis, can play an important role in reducing heavy metal uptake and consequently diminishing metal toxins in plants [47]. Furthermore, extension of apoplastic barriers, such as development of endodermis under metal stresses, are among important mechanisms to prevent the translocation of cadmium to aerial parts [48]. This matter has been reported in the rice plant [49]. Similar results obtained from Qiong Zhong's study on* Avicennia* reported that silicon with expanding and improved apoplastic barriers in roots can reduce Cd uptake in plants [47].

### 2.2. Mechanism Two: Changes in pH Value in Soil and Plant Culture

The pH value plays an important role in bioavailability and mobility of heavy metals in soil and culture [50–54]. One of the main mechanisms of silicon amelioration is the role of silicon in changing soil and growth medium's pH. Silicon compounds, like biosolids, increase the pH value so that this increase can improve the absorption of silicon. On the other hand, it can lead to an immobilization and unavailability of heavy metals, such as Cd, in plants [16] and also to a reduction of heavy metal bioavailability. Organic materials and pH exist in soil, including soil sodium metasilicate or alkaline pH, playing an important role in the reduction of metal availability in soil and consequently amelioration of metal toxicity to plants [39]. The results of [55] show that reducing the toxicity of aluminum metal ions by changing the pH in a medium can be one of the external mechanisms in amelioration of Al by silicon in soybeans. This can later lead to the unavailability and precipitation of AL [30]. Moreover, silicon can facilitate metal transport in plants. As a result, silicon with the formation of hydroxy aluminum silicate complexes in shoots can increase the transportation from root to shoot. Kopittke et al. (2017) reported that detoxification of aluminum by silicon is related to the formation of hydroxy aluminum silicates in roots [56]. However, this formation depends on pH [57], so that the formation of hydroxy aluminosilicate reduces the pH value to less than 4.0 [58].

It is seen that, in exposure to metal stress, such as extreme concentration of Al, silicon accumulates in cell walls and leads to a reduction in Al toxicity in the apoplasm. The result of one experiment showed that Al with formation of hydroxyl aluminum silicates in root apoplast can convert the Al to a nontoxic form in the apoplast and ameliorate the Al toxicity in the plant root. This issue indicates the silicon mechanism to reduce metal toxicity in the apoplast. The rate of HAS (hydroxy aluminum species) efficiency depends on the enhancement of pH and high concentration of Al and Si [58]. The pH value is an important factor in the formation of HAS. Al toxicity often occurs in low pH, and HAS formation does not have enough efficiency in low pH (<5) [58].

### 2.3. Mechanism Three: Formation of Si Heavy Metals

Silicon, in the first step, detoxifies the heavy metals in plants with (1) solution chemistry mechanism (i.e., making complexes with heavy metals) and (2) planta mechanism [1, 30, 59] (i.e., stimulating the organic acid exudate from plants to chelate metals ions) [1, 30, 59]. In the solution chemistry mechanism, silicon creates a compound by forming silicates and oxides with heavy metals, which is caused by the unavailability of Si in plants [59]. For instance, Si, in a complex formation of Al-Si, decreases the toxicity of Al^3+^ [30, 56]. Additionally, it decreases the Al availability [55] and reduces the free Al by the formation of the aluminum silicate compound in plant cells [60]. The plant cell wall has an important site in colocalized Al-Si in hypodermal and epidermal cells [61], which is shown to be a major site for accumulation of silicon to make wall-bound organosilicon compounds [62]. Investigating* Minuartia verna*, Neumann et al. expressed that formation of Zn-silicate precipitated in the leaf epidermis acts as an important pathway for Zn detoxification [63]. There are different ideas regarding the impact of silicon on the cell wall. In some experiments on the cell wall, the results showed that silicon could not significantly reduce the Al concentration, but they indicated an exchange enhancement in Al-cell wall binding. Therefore, it was concluded that silicon decreases the Al-cell wall binding in the apoplast [58]. There are, however, other researchers who attributed this factor to the reduction of alumina biologic activities; they believe that Si could not reduce Al concentration in the cell wall, but Si can decrease the ability of Al biologic activity in the cell wall. This is assumed as a factor in the reduction of aluminum efficiency in connection with the cell wall, which reduces the toxicity of aluminum. Prabagar et al. (2011) demonstrated that degradation of free Al by silicon in the cell wall can be an important factor to protect the plant cells in* P. abies* [60] so that the reduction of Al biological availability, hydroxy aluminum species, and silicic acid is key in the formation of HAS in low pH [30]. Silicon involved in the translocation of the cell wall is one of the crucial mechanisms in the reduction of heavy metal toxicity in plants [13, 64]. One experiment conducted by Gunes et al. (2007) showed that silicon limited baron translocation from root to shoot in spinach [65]. Formation of Si heavy metals in ultrastructures revealed the role of Si in heavy metal transport. The results of another experiment reported that, in* Cardaminopsis* sp., silicon actively contributes to the transportation of Zn to vacuoles. Through this process, Zn precipitates to the cytoplasm with silicate formation. The formation of Zn-silicate during such a fast process degrades to SiO_2_ and Zn immediately. Then, Zn is transferred to the vacuole, and SiO_2_ precipitates in the cytoplasm [17]. The other pathway is related to plasma member and tonoplast forming pinocytotic vesicles in which Zn is directly transferred from extracellular fractions to vacuoles [39]. This is known as a compartmentation mechanism in plants. In both cases, the formation of Si heavy metals has a vital role in the digestion and precipitation of metal ions. It can be concluded that silicon can reduce the heavy metal mobility with chemical interaction mechanisms, such as formation of Si heavy metal [39].

### 2.4. Mechanism Four: Stimulation of Enzyme Activities

Metals ions, with distribution in photosynthesis electron transfer (Phet) and reduction of net photosynthesis (Pn), may lead to a severe impairment in photosynthetic metabolism [66]. Severe impairment in photosynthetic metabolism is expressed as an important factor in the generation of ROS derivatives, such as H_2_O_2_, O^−2^, and OH [67] in chloroplasts, mitochondria, and plasma membranes [68], which is a primary response of plants to oxidative stress [69, 70]. An ROS compound is divided into two categories: (1) nonradical molecules, such as singlet oxygen (1O_2_) and hydrogen peroxide (H_2_O_2_), and (2) free radicals. Free radicals include hydroxyl radical (•OH), superoxide anion (O2•−), alkoxy radical, perhydroxyl radical (HO_2_), reactive molecules, and ions. Chloroplasts and mitochondria are major colonies in generating O_2_ and O^−2^ [68, 70]. The primary location in the plant for generating ROS includes the reaction center of PS1 and PS11 in chloroplast thylakoid membranes. Generation of ROS usually occurs with the excess of photons (p) in environmental changes (environmental stress) when there is an extra dose of CO_2_ assimilation (A) (P > A) [71]. ROS derivatives increase oxidative stress in plants, which can lead to an increase in MDA and lipid peroxidation, disturbance in enzyme activity and amino acids in cells, and protein oxidation [68, 72, 73]. Thus, damaging impacts of ROS can be summarized as follows:

- Morphological impacts, including (1) decreasing of root and shoot growth and (2) leaf curling
- Biochemical impacts, such as (1) membrane damaging, (2) permeability, and (3) protein structure
- Physiological impacts, such as (1) chlorosis, (2) photosynthesis, and (3) metal uptake [74, 75].

Silicon accumulation in different plant tissues, such as root, stem, leaves, and hulls, can preserve the plant from abiotic and biotic stresses [76]. Plants' major defense mechanisms to adjust to heavy metal stress and to protect plant cells from oxidative stress are scavenging free radicals by ROS. ROS stimulates enzyme activities, either antioxidant enzymes or nonenzyme activities [77–80]. Antioxidant enzymes and nonenzymes include superoxide dismutase (SOD), peroxidase (POD), catalase (CAT), guaiacol peroxidase (GPX), ascorbate peroxidase (APX), glutathione reductase (GR) [65, 71, 81, 82], ascorbic acid (AA), flavonoids, reduced glutathione, *α*-tocopherol, carotenoids, and osmolyte proline [83, 84]. They can scavenge ROS in plants with some chemical cycles, such as ascorbate-glutathione, water-water, and peroxisomal glutathione peroxidase. And these chemical cycles are in intercell organs including cytosol, mitochondria, chloroplast, apoplast, and the peroxisomes [85–87]. They can maintain plant integrity against metal stress inside mitochondria, nuclei, and chloroplasts [28, 88]. In this case, SOD converts superoxide anion to peroxide [63]. CAT catalyzes the conversion of H_2_O_2_ to water and O_2_ [89, 90]. Ascorbic oxidase majorly contributes to the regulation of GR and NADPH [91]. Glutathione, which shows intracellular redox potential and ascorbate, would then be involved in cytoplasmic and apoplastic signaling [92, 93]. In terms of antioxidant activity, silicon leads to stimulation of antioxidant enzyme activity in plant growth under heavy metal stress [37]. This issue has been shown in many plants under different metal stressors, including soybean [94], barley [65], rice [95],* A. thaliana* [96], cotton [7], banana [97],* Brassica chinensis* L [28], peanut [35], and ramie [98]. For instance, the results obtained from* Ajuga multiflora* indicated that the medium with extra dose of silicon to MS can increase shoot regeneration by increasing the antioxidant enzyme activities, such as SOD, POD, APX, and CAT [99]. However, other sources indicated that silicon can impact the Mn uptake by root and reduce Mn concentration in cucumber [100]. A similar result was reported for sorghum [101]. In another experiment on rice exposed to extreme doses of Zn, it is shown that Si application increases the antioxidant activities, such as SOD, CAT, and APX, while reducing the H_2_O_2_ and MDA content [95]. Feng et al. indicated that an extra dose of Si applied in cucumber and exposed to manganese toxicity increases antioxidant and nonantioxidant enzyme activities, including SOD, APX, DHAR, GR, ascorbate, and GSH and decreases the lipid peroxidation [102]. Additionally, Song et al. obtained the same result in a study on cucumber exposed to Mn [95]. In general, silicon plays an important role in increasing the antioxidants in plants. However, the efficiency of this mechanism relates to the concentration of heavy metals so that in a high dose of metal toxicity antioxidant activity may not work well [13].

#### 2.4.1. Silicon via Ascorbate

Ascorbate, as a regulator with small molecular weight, can regulate cell processes, including those through cell cycle, those during cell expansion, and senescence [103]. The main site of ascorbate is located in the mesophyll cells of leaves with 40% storage in the chloroplast, which is often decreased in stress conditions [70]. Ninety percent of AsA ascorbate is localized in cell cytoplasm, known as a frontier line in the interference with external oxidant damage [104]. It plays an important role in removing H_2_O_2_ from water [105]. Additionally, reduced glutathione (GSH) is an important component for the formation of ascorbate-GSH (AsA-GSH), which consists of the bench of the enzyme, including GSH sulfotransferases (GSTs), glutathione peroxidase (GPX), and glutathione reductase (GR) [106]. GSH, as a biothiol tripeptide, plays a vital role in cell tolerance to metal stresses using two pathways: (1) antioxidant and phytochelatins (PCs) regulating redox imbalance and (2) reducing the concentration of free ion cellular, respectively [107, 108], that is important to the amelioration of heavy metal stress as the antioxidant [109, 110]. Glutathione peroxidase (GPX, EC 1.11.1.9.), as one peroxidase enzyme, plays an important role in the degradation of ROS compounds in cytoplasm and apoplast area [68] and also biosynthesis of lignin [111]. GPX can scavenge ROS from intra- and extracellular media [112]. Additionally, it would scavenge H_2_O_2_ by reducing the glutathione and regenerating GSSG using glutathione reductase (GR) [70]. Ascorbate peroxidase (APX) (1.1.11.1) is known to be the indicator of H_2_O_2_ amounts in chloroplasts and cytosol, which can be used to degrade the ascorbic acid [105]. APX in the ascorbate-glutathione cycle degrades H_2_O_2_ to water (as a cofactor) [70, 113] which can raise its activity with some enzyme functions, such as SOD, CAT, and GSH reductase [105]. APX is the main form of AsA-GSH cycle which has the ability to scavenge H_2_O_2_ to H_2_O and oxygen with MDHA molecular [105]. The ability to scavenge H_2_O_2_ in APX is much stronger than CAT [105, 114]. The role of APX in cell protection is crucial and can cover plant cells with five different isoforms, including cytosolic form (cAPX), chloroplast stromal soluble form (s APX), and thylakoid (t APX) glyoxysome membrane form (gmAPX) [105]. The role of silicon is to increase the APX, GSH, and AsA activity under heavy metal stress conditions. Silicon application also has a positive impact on the ascorbate-glutathione cycle and can improve it by increasing APX [28]. Thus, it can be concluded that silicon indirectly associates with the degradation of ROS compounds, such as H_2_O_2_ and OH to detoxify plant toxicity, and consequently can increase the plant tolerance to metal stress.

#### 2.4.2. Silicon via Glutathione

Glutathione, as a redox buffer, plays an important role in the antioxidant mechanisms to scavenge ROS by preserving the balance of cellular redox [115]. Glutathione reductase (GR, EC, 1.6.4.2) is a compound containing disulfide groups. It is categorized as flavoenzymes, which can work with some mechanisms, including catalyzing and oxidizing flavin with NADH and disulfide. It also interchanges some reactions by GSSG degradation in disulfate [116], which has an important role in the synthesis of phytochelatins and is an essential factor in sequestering heavy metals [117]. Additionally, it has the ability to scavenge ROS by converting it to sulfhydryl form GSH through catalyzing glutathione disulfide [118], which has a major role in the control of H_2_O_2_ levels [119]. Glutathione reductase occurs during the photosynthetic process for scavenging and degradation of H_2_O_2_ [120, 121] and was often localized in chloroplasts. However, a small amount of that was also found in mitochondria and cytosol, which play the catalyst role in ASH-GSH cycle by mechanisms of degradation and regeneration of GSH [122]. GSH plays an important role in the cell system through certain mechanisms such as the regulation of the sulfhydryl (-SH) group and GSTs [123]. In addition, it is known to be one of the important antioxidant and redox buffers and has an important role in cell division [123]. GR and APX, with the ascorbate-glutathione cycle, play an important role in scavenging ROS by degrading H_2_O_2_, so that ascorbate converts H_2_O_2_ to H_2_O and GR in the first line of this pathway and continues degrading H_2_O_2_ to reduced glutathione level in the last step [71, 124].

#### 2.4.3. Superoxide Dismutase

The soluble enzyme dismutase has an important duty in the dismutation of O^−2^ to O_2_ and H_2_O_2_ [125]. It also plays a vital role in cell protection. In the case of heavy metal toxicity, superoxide dismutase (SOD) with enzyme code (EC 1.15.1.1) is known to be the first line in the detoxification of ROS compound. Firstly the enzyme causes a dismutation of O^−2^ and secondly it reduces the possibility of OH formation [125]. The dismutation reaction is conducted by three types of formations to use different metals as cofactors in SOD, including manganese (Mn-SOD), iron (Fe-SOD), and copper/zinc (Cu/Zn-SOD) [126]. The SOD site, in a plant cell, can be located in the chloroplast, mitochondria, cytosol, or peroxisomes. More precisely, the sites of these three types of formations are normally in peroxisomes; however, their specific sites are manganese in mitochondria, iron in the chloroplast, and copper/zinc in glyoxysomes, chloroplast, and cytosol [127, 128].

#### 2.4.4. Catalase

In peroxisomes and photorespiration, catalase acts as a dismutation factor in scavenging H_2_O_2_ to oxygen and H_2_O through the process of *β*-oxidation of fatty acids. Oxidation has a vital role in the digestion of the ROS components especially for H_2_O_2_ [129, 130]. One molecule of CAT can catalyze two molecules of H_2_O_2_ to water and oxygen [131]. Additionally, CAT can degrade some hydroperoxide groups, including methyl hydrogen peroxide (MeOH). By controlling the H_2_O_2_ compound, CAT preserves cell walls from lipid production and membrane damage. It is also involved in photosynthesis and prevents chlorophyll degradation [132].

## 3. Conclusion

Silicon is the second most abundant element in soil and the earth's crust. It cannot be found as a free element in soil and always appears as a combination of oxygen and silicate and other elements, which can be used in plants as silicic acid, Si(OH)_4_. Silicon can be considered a quasi-essential element to increase plant growth and development and to reduce the abiotic and biotic stresses in plants through different mechanisms. It also plays a positive role in increasing the plant's resistance against stress, which can be achieved by silicon accumulation in plant parts, including roots stem, leaves, and hulls.

Silicon is generally used in plant protection processes against heavy metals in two mechanisms: avoidance and tolerance. Avoidance mechanisms include silicon reducing heavy metal uptake and availability by increasing the soil pH. It can additionally chelate-heavy metal compounds with root exudates, such as phenolics and organic acids, or decrease the translocation of heavy metals in the plant. However, in tolerance mechanisms, silicon elevates heavy metal stress with various mechanisms, including compartmentalizing heavy metals into cell walls and vacuoles, increasing enzyme antioxidant and nonenzyme antioxidant activity, limiting transportation in plants, homogeneously distributing metals in the leaf surface, and chelating or making a heavy metal barrier to reduce translocation in plants. The pH value mechanism caused by silicon is one of the important mechanisms for amelioration of heavy metals. Having additional silicon in the soil causes the pH value to increase. It can be vital for the immobility of heavy metals, may decrease heavy metal uptake, and can finally reduce the precipitation of silicon and heavy metals. Reduction of heavy metal uptake can be done in two ways: chemical or physical pathways. In the first way, stimulating the root exudate, silicon leads to an increase in chelating heavy metals, which can then reduce the ion uptake by plants. In the other case, plasmids in root cells prevent the translocation of heavy metals from root to shoot with building barriers. This can be counted as a physical mechanism to reduce heavy metal uptake in plants. The terms “chemical solution” and “planta” in silicon mechanisms are very important; “planta” expresses the increase in organic acid exuded from plants for chelation purposes of metals ions, as previously discussed. However, “chemical solutions” often occur in plant cells; they have this ability to initiate a silicon-to-metals ion formation and make a new compound of the silicon-heavy metal, which can cause the precipitation of heavy metal and silicon in the space between cells and phytoliths. The impacts of silicon on antioxidant enzymes' and nonenzymes' activities are considered one of the important mechanisms to reduce the negative effects of heavy metals in plants. This can effectively protect plant cells from oxidative stress and scavenging free radicals caused by ROS to stimulate both antioxidant enzymes' and nonenzymes' activities, such as superoxide dismutase (SOD), peroxidase (POD), catalase (CAT), guaiacol peroxidase (GPX), ascorbate peroxidase (APX), glutathione reductase (GR), ascorbic acid (AA), flavonoids, reduced glutathione, *α*-tocopherol, carotenoids, and osmolyte proline. Antioxidants can scavenge ROS in plants with some chemical cycles, such as ascorbate-glutathione, water-water, and peroxisomal glutathione peroxidase. And these chemical cycles are in plant intercell organs, including cytosol, mitochondria, chloroplasts, apoplast, and peroxisomes. Another mechanism related to silicon mediation under heavy metal stress is gene expression. Physiologic alteration in plants related to a change in gene expression and changes in plant structures causes amelioration of heavy metals. This topic requires more research to identify mechanisms and thus will be followed by the authors in future research.

This article attempted to highlight the major mechanisms of silicon in reducing and ameliorating heavy metals in plants. Additionally, it tried to address the role of genes and intracellular organs and nuclei in plants.

## References

[1] Sahebi M., Hanafi M. M., Siti Nor Akmar A. (2015). Importance of silicon and mechanisms of biosilica formation in plants. *BioMed Research International*.

[2] Cooke J., Leishman M. R. (2011). Is plant ecology more siliceous than we realise?. *Trends in Plant Science*.

[3] Coskun D., Britto D. T., Huynh W. Q., Kronzucker H. J. (2016). The role of silicon in higher plants under salinity and drought stress. *Frontiers in Plant Science*.

[4] Neu S., Schaller J., Dudel E. G. (2017). Silicon availability modifies nutrient use efficiency and content, C:N:P stoichiometry, and productivity of winter wheat (Triticum aestivum L.). *Scientific Reports*.

[5] Liang Y., Nikolic M., Bélanger R., Gong H., Song A. (2015). Effect of silicon on crop growth, yield and quality. *Silicon in Agriculture*.

[6] Cuong T. X., Ullah H., Datta A., Hanh T. C. (2017). Effects of silicon-based fertilizer on growth, yield and nutrient uptake of rice in tropical zone of Vietnam. *Rice Science*.

[7] Farooq M. A., Ali S., Hameed A., Ishaque W., Mahmood K., Iqbal Z. (2013). Alleviation of cadmium toxicity by silicon is related to elevated photosynthesis, antioxidant enzymes; suppressed cadmium uptake and oxidative stress in cotton. *Ecotoxicology and Environmental Safety*.

[8] Song A., Li P., Fan F., Li Z., Liang Y. (2014). The effect of silicon on photosynthesis and expression of its relevant genes in rice (Oryza sativa L.) under high-zinc stress. *PLoS ONE*.

[9] Xie Z., Song F., Xu H., Shao H., Song R. (2014). Effects of silicon on photosynthetic characteristics of maize (Zea mays L.) on alluvial soil. *The Scientific World Journal*.

[10] Tripathi D. K., Singh V. P., Gangwar S., Prasad S. M., J.N.Maurya J. N., Chauhan D. K., Ahmad P., Wani M. R., Azooz M. M., Tran L. S. P. (2014). Role of silicon in enrichment of plant nutrients and protection from biotic and abiotic stresses. *Improvement of Crops in the Era of Climatic Changes*.

[11] Ma J. F., Yamaji N., Mitani-Ueno N. (2011). Transport of silicon from roots to panicles in plants. *Proceedings of the Japan Academy Series B: Physical and Biological Sciences*.

[12] Van Bockhaven J., De Vleesschauwer D., Höfte M. (2013). Towards establishing broad-spectrum disease resistance in plants: silicon leads the way. *Journal of Experimental Botany*.

[13] Adrees M., Ali S., Rizwan M. (2015). Mechanisms of silicon-mediated alleviation of heavy metal toxicity in plants: a review. *Ecotoxicology and Environmental Safety*.

[14] Huang Y. Z., Zhang W. Q., Zhao L. J. (2012). Silicon enhances resistance to antimony toxicity in the low-silica rice mutant, lsi1. *Chemistry and Ecology*.

[15] Singh V. P., Tripathi D. K., Kumar D., Chauhan D. K. (2011). Influence of exogenous silicon addition on aluminium tolerance in rice seedlings. *Biological Trace Element Research*.

[16] Liang Y., Nikolic M., Bélanger R., Gong H., Song A. (2015). Silicon-mediated tolerance to metal toxicity. *Silicon in Agriculture: From Theory to Practice*.

[17] Tubana B. T., Heckman J. R., Rodrigues F. A., Datnoff L. E. (2015). *Silicon and Plant Diseases*.

[18] Liang Y., Sun W., Zhu Y., Christie P. (2007). Mechanisms of silicon-mediated alleviation of abiotic stresses in higher plants: a review. *Environmental Pollution*.

[19] Ma J. F., Yamaji N. (2008). Functions and transport of silicon in plants. *Cellular and Molecular Life Sciences*.

[20] Martirosyan D. (2006). Functional food for cronic diseases. *Protective Role of Silicon in Living Systems, Books › New, Used & Rental Textbooks › Medicine & Health Sciences*.

[21] Sivanesan I., Park S. W. (2014). The role of silicon in plant tissue culture. *Frontiers in Plant Science*.

[22] Manivannan A., Ahn Y.-K. (2017). Silicon regulates potential genes involved in major physiological processes in plants to combat stress. *Frontiers in Plant Science*.

[23] Chen D., Cao B., Wang S. (2016). Silicon moderated the K deficiency by improving the plant-water status in sorghum. *Scientific Reports*.

[24] Liu Y., Wang X., Zeng G. (2007). Cadmium-induced oxidative stress and response of the ascorbate-glutathione cycle in Bechmeria nivea (L.) Gaud. *Chemosphere*.

[25] Piperno D. R. (2006). *Phytoliths a Comprehensive Guide Archaeologist and Paleoecologists*.

[26] Hartley S. E., DeGabriel J. L. (2016). The ecology of herbivore-induced silicon defences in grasses. *Functional Ecology*.

[27] da Cunha K. P. V., do Nascimento C. W. A. (2009). Silicon effects on metal tolerance and structural changes in Maize *(Zea mays* L.) grown on a cadmium and zinc enriched soil. *Water, Air, & Soil Pollution*.

[28] Song A., Li Z., Zhang J., Xue G., Fan F., Liang Y. (2009). Silicon-enhanced resistance to cadmium toxicity in Brassica chinensis L. is attributed to Si-suppressed cadmium uptake and transport and Si-enhanced antioxidant defense capacity. *Journal of Hazardous Materials*.

[29] Liang Y., Yang C., Shi H. (2001). Effects of silicon on growth and mineral composition of barley grown under toxic levels of aluminum. *Journal of Plant Nutrition*.

[30] Pontigo S., Godoy K., Jiménez H., Gutiérrez-Moraga A., Mora M. D. L. L., Cartes P. (2017). Silicon-mediated alleviation of aluminum toxicity by modulation of al/si uptake and antioxidant performance in ryegrass plants. *Frontiers in Plant Science*.

[31] Guntzer F., Keller C., Meunier J.-D. (2012). Benefits of plant silicon for crops: a review. *Agronomy for Sustainable Development*.

[32] Gu H.-H., Qiu H., Tian T. (2011). Mitigation effects of silicon rich amendments on heavy metal accumulation in rice (Oryza sativa L.) planted on multi-metal contaminated acidic soil. *Chemosphere*.

[33] Kim Y.-H., Khan A. L., Waqas M., Lee I.-J. (2017). Silicon regulates antioxidant activities of crop plants under abiotic-induced oxidative stress: a review. *Frontiers in Plant Science*.

[34] Savvas D., Ntatsi G. (2015). Biostimulant activity of silicon in horticulture. *Scientia Horticulturae*.

[35] Shi G., Cai Q., Liu C., Wu L. (2010). Silicon alleviates cadmium toxicity in peanut plants in relation to cadmium distribution and stimulation of antioxidative enzymes. *Plant Growth Regulation*.

[36] Kaya C., Tuna A. L., Sonmez O., Ince F., Higgs D. (2009). Mitigation effects of silicon on maize plants grown at high zinc. *Journal of Plant Nutrition*.

[37] Ma J. F., Yamaji N. (2006). Silicon uptake and accumulation in higher plants. *Trends in Plant Science*.

[38] Ye J., Yan C., Liu J., Lu H., Liu T., Song Z. (2012). Effects of silicon on the distribution of cadmium compartmentation in root tips of Kandelia obovata (S., L.) Yong. *Environmental Pollution*.

[39] Wu J.-W., Shi Y., Zhu Y.-X., Wang Y.-C., Gong H.-J. (2013). Mechanisms of enhanced heavy metal tolerance in plants by silicon: a review. *Pedosphere*.

[40] Doncheva S., Poschenrieder C., Stoyanova Z., Georgieva K., Velichkova M., Barceló J. (2009). Silicon amelioration of manganese toxicity in Mn-sensitive and Mn-tolerant maize varieties. *Environmental and Experimental Botany*.

[41] Gong H., Zhu X., Chen K., Wang S., Zhang C. (2005). Silicon alleviates oxidative damage of wheat plants in pots under drought. *Journal of Plant Sciences*.

[42] Rizwan M., Meunier J.-D., Miche H., Keller C. (2012). Effect of silicon on reducing cadmium toxicity in durum wheat (Triticum turgidum L. cv. Claudio W.) grown in a soil with aged contamination. *Journal of Hazardous Materials*.

[43] Sommer M., Kaczorek D., Kuzyakov Y., Breuer J. (2006). Silicon pools and fluxes in soils and landscapes—a review. *Journal of Plant Nutrition and Soil Science*.

[44] Currie H. A., Perry C. C. (2007). Silica in plants: biological, biochemical and chemical studies. *Annals of Botany*.

[45] Ma J. F. (2004). Role of silicon in enhancing the resistance of plants to biotic and abiotic stresses. *Soil Science & Plant Nutrition*.

[46] Ahmad P., Tripathi D. K., Singh S., Chauhan D. K., Dubey N. K., Prasad R., Ahmad P. (2016). Silicon as a beneficial element to combat the adverse effect of drought in agricultural crops. *Water Stress and Crop Plants: A Sustainable Approach*.

[47] Zhang Q., Yan C., Liu J. (2013). Silicon alleviates cadmium toxicity in Avicennia marina (Forsk.) Vierh. seedlings in relation to root anatomy and radial oxygen loss. *Marine Pollution Bulletin*.

[48] Lux A., Martinka M., Vaculík M., White P. J. (2011). Root responses to cadmium in the rhizosphere: a review. *Journal of Experimental Botany*.

[49] Krishnamurthy P., Ranathunge K., Nayak S., Schreiber L., Mathew M. K. (2011). Root apoplastic barriers block Na^+^ transport to shoots in rice (Oryza sativa L.). *Journal of Experimental Botany*.

[50] Zhao X.-L., Masaihiko S. (2007). Amelioration of cadmium polluted paddy soils by porous hydrated calcium silicate. *Water, Air, & Soil Pollution*.

[51] Violante A., Cozzolino V., Perelomov L., Caporale A. G., Pigna M. (2010). Mobility and bioavailability of heavy metals and metalloids in soil environments. *Soil Science & Plant Nutrition*.

[52] Adamczyk-Szabela D., Markiewicz J., Wolf W. M. (2015). Heavy metal uptake by herbs. IV. influence of soil pH on the content of heavy metals in Valeriana officinalis L. *Water, Air, & Soil Pollution*.

[53] Caporale A. G., Violante A. (2016). Chemical processes affecting the mobility of heavy metals and metalloids in soil environments. *Current Pollution Reports*.

[54] Lu L., Liu G., Wang J., Wu Y. (2017). Bioavailability and mobility of heavy metals in soil in vicinity of a coal mine from Huaibei, China. *Human and Ecological Risk Assessment*.

[55] Baylis A. D., Gragopoulou C., Davidson K. J., Birchall J. D. (1994). Effects of silicon on the toxicity of aluminium to soybean. *Communications in Soil Science & Plant Analysis*.

[56] Kopittke P. M., Gianoncelli A., Kourousias G., Green K., McKenna B. A. (2017). Alleviation of Al toxicity by Si is associated with the formation of Al-Si complexes in root tissues of sorghum. *Frontiers in Plant Science*.

[57] Povar I., Spinu O. (2014). The role of hydroxy aluminium sulfate minerals in controlling Al 3^+^ concentration and speciation in acidic soils. *Central European Journal of Chemistry*.

[58] Wang Y., Stass A., Horst W. J. (2004). Apoplastic binding of aluminum is involved in silicon-induced amelioration of aluminum toxicity in maize. *Plant Physiology*.

[59] Exley C. (2015). A possible mechanism of biological silicification in plants. *Frontiers in Plant Science*.

[60] Prabagar S., Hodson M. J., Evans D. E. (2011). Silicon amelioration of aluminium toxicity and cell death in suspension cultures of Norway spruce (Picea abies (L.) Karst.). *Environmental and Experimental Botany*.

[61] Horst W. J., Wang Y., Eticha D. (2010). The role of the root apoplast in aluminium-induced inhibition of root elongation and in aluminium resistance of plants: a review. *Annals of Botany*.

[62] Liu J., Ma J., He C. (2013). Inhibition of cadmium ion uptake in rice (Oryza sativa) cells by a wall-bound form of silicon. *New Phytologist*.

[63] Neumann D., Nieden U. Z., Schwieger W., Leopold I., Lichtenberger O. (1997). Heavy metal tolerance of minuartia verna. *Journal of Plant Physiology*.

[64] Guerriero G., Hausman J.-F., Legay S. (2016). Silicon and the plant extracellular matrix. *Frontiers in Plant Science*.

[65] Gunes A., Inal A., Bagci E. G., Coban S., Pilbeam D. J. (2007). Silicon mediates changes to some physiological and enzymatic parameters symptomatic for oxidative stress in spinach (Spinacia oleracea L.) grown under B toxicity. *Scientia Horticulturae*.

[66] Yruela I. (2013). Transition metals in plant photosynthesis. *Metallomics*.

[67] Pinto S. D. S., de Souza A. E., Oliva M. A., Pereira E. G. (2016). Oxidative damage and photosynthetic impairment in tropical rice cultivars upon exposure to excess iron. *Scientia Agricola*.

[68] Sharma P., Jha A. B., Dubey R. S., Pessarakli M. (2012). Reactive oxygen species, oxidative damage, and antioxidative defense mechanism in plants under stressful conditions. *Journal of Botany*.

[69] Asgher M., Per T. S., Anjum S. (2017). Contribution of glutathione in heavy metal stress tolerance in plants. *Reactive Oxygen Species and Antioxidant Systems in Plants: Role and Regulation under Abiotic Stress*.

[70] Gill S. S., Tuteja N. (2010). Reactive oxygen species and antioxidant machinery in abiotic stress tolerance in crop plants. *Plant Physiology and Biochemistry*.

[71] Asada K. (2006). Production and scavenging of reactive oxygen species in chloroplasts and their functions. *Plant Physiology*.

[72] You J., Chan Z. (2015). Ros regulation during abiotic stress responses in crop plants. *Frontiers in Plant Science*.

[73] Krishnamurthy A., Rathinasabapathi B. (2013). Oxidative stress tolerance in plants: novel interplay between auxin and reactive oxygen species signaling. *Plant Signaling and Behavior*.

[74] Yadav S. K. (2010). Heavy metals toxicity in plants: an overview on the role of glutathione and phytochelatins in heavy metal stress tolerance of plants. *South African Journal of Botany*.

[75] Gupta D. K., Corpas F. J., Palma J. M., Hirt H. (2013). *Heavy Metal Stress in Plants, Plant Stress Biolog*.

[76] Ma J. F., Yamaji N. (2015). A cooperative system of silicon transport in plants. *Trends in Plant Science*.

[77] Arif N., Yadav V., Singh S. (2016). Assessment of antioxidant potential of plants in response to heavy metals. *Plant Responses to Xenobiotics*.

[78] Pang C., Wang B., Lüttge U., Beyschlag W., Murata J. (2008). Oxidative stress and salt tolerance in plants. *Progress in Botany*.

[79] Kumar M., Kumari P., Gupta V., Anisha P. A., Reddy C. R. K., Jha B. (2010). Differential responses to cadmium induced oxidative stress in marine macroalga Ulva lactuca (Ulvales, Chlorophyta). *BioMetals*.

[80] Lin L., Zhou W., Dai H., Cao F., Zhang G., Wu F. (2012). Selenium reduces cadmium uptake and mitigates cadmium toxicity in rice. *Journal of Hazardous Materials*.

[81] Ahmad I. Z., Ahmad A., Mabood A., Tabassum H., Khan M., Khan N. (2017). Effects of different metal stresses on the antioxidant defense systems of medicinal plants. *Reactive Oxygen Species and Antioxidant Systems in Plants: Role and Regulation under Abiotic Stress*.

[82] Racchi M. (2013). Antioxidant defenses in plants with attention to prunus and citrus spp. *Antioxidants*.

[83] Rao N. K. S., Shivashankara K. S., Laxman R. H. (2016). *Abiotic Stress Physiology of Horticultural Crops*.

[84] Anjum N. A., Khan N. A., Sofo A., Baier M., Kizek R. (2016). Editorial: redox homeostasis managers in plants under environmental stresses. *Frontiers in Environmental Science*.

[85] Foyer C. H., Noctor G. (2011). Ascorbate and glutathione: the heart of the redox hub. *Plant Physiology*.

[86] Czarnocka W., Karpiński S. (2018). Friend or foe? Reactive oxygen species production, scavenging and signaling in plant response to environmental stresses. *Free Radical Biology and Medicine*.

[87] Hasanuzzaman M., Hossain M. A., Teixeira da Silva J. A., Fujita M., Bandi V., Shanker A. K., Shanker C., Mandapaka M. (2012). Plant response and tolerance to abiotic oxidative stress: antioxidant defense is a Key factor. *Crop Stress and Its Management: Perspectives and Strategies*.

[88] Nwugo C. C., Huerta A. J. (2008). Effects of silicon nutrition on cadmium uptake, growth and photosynthesis of rice plants exposed to low-level cadmium. *Plant and Soil*.

[89] Das K., Roychoudhury A. (2014). Reactive oxygen species (ROS) and response of antioxidants as ROS-scavengers during environmental stress in plants. *Frontiers in Environmental Science*.

[90] Singh V. P., Singh S., Tripathi D. K., Prasad S. M., Chauhan D. K. (2017). *Reactive Oxygen Species in Plants: Boon Or Bane - Reactive Oxygen Species in Plants: Boon Or Bane - Revisiting the Role of ROS*.

[91] Sies H. (2007). Total antioxidant capacity: appraisal of a concept. *Journal of Nutrition*.

[92] Zechmann B. (2014). Compartment-specific importance of glutathione during abiotic and biotic stress. *Frontiers in Plant Science*.

[93] Noctor G., Mhamdi A., Chaouch S. (2012). Glutathione in plants: an integrated overview. *Plant, Cell & Environment*.

[94] Miao B.-H., Han X.-G., Zhang W.-H. (2010). The ameliorative effect of silicon on soybean seedlings grown in potassium-deficient medium. *Annals of Botany*.

[95] Song A., Li P., Li Z., Fan F., Nikolic M., Liang Y. (2011). The alleviation of zinc toxicity by silicon is related to zinc transport and antioxidative reactions in rice. *Plant and Soil*.

[96] Khandekar S., Leisner S. (2011). Soluble silicon modulates expression of Arabidopsis thaliana genes involved in copper stress. *Journal of Plant Physiology*.

[97] Li L., Zheng C., Fu Y., Wu D., Yang X., Shen H. (2012). Silicate-mediated alleviation of Pb toxicity in banana grown in Pb-contaminated soil. *Biological Trace Element Research*.

[98] Tang H., Liu Y., Gong X. (2015). Effects of selenium and silicon on enhancing antioxidative capacity in ramie (Boehmeria nivea (L.) Gaud.) under cadmium stress. *Environmental Science and Pollution Research*.

[99] Sivanesan I., Jeong B. R. (2014). Silicon promotes adventitious shoot regeneration and enhancessalinity tolerance of *Ajuga multiflora* bunge by altering activityof antioxidant enzyme. *The Scientific World Journal*.

[100] Maksimović J. D., Mojović M., Maksimović V., Römheld V., Nikolic M. (2012). Silicon ameliorates manganese toxicity in cucumber by decreasing hydroxyl radical accumulation in the leaf apoplast. *Journal of Experimental Botany*.

[101] Galvez L., Clark R. B., Gourley L. M., Maranville J. W. (2008). Effects of silicon on mineral composition of sorghum grown with excess manganese. *Journal of Plant Nutrition*.

[102] Feng J. p., Shi Q. h., Wang X. f. (2009). Effect of exogenoud silicon on photosyntesis capacity and antioxidant anzyme activity in choloroplast of cucumber seedling under excess maneges. *Agricultural Sciences in China*.

[103] Szarka A., Bánhegyi G., Asard H. (2013). The inter-relationship of ascorbate transport, metabolism and mitochondrial, plastidic respiration. *Antioxidants & Redox Signaling*.

[104] Akram N. A., Shafiq F., Ashraf M. (2017). Ascorbic acid-a potential oxidant scavenger and its role in plant development and abiotic stress tolerance. *Frontiers in Plant Science*.

[105] Caverzan A., Passaia G., Rosa S. B., Ribeiro C. W., Lazzarotto F., Margis-Pinheiro M. (2012). Plant responses to stresses: role of ascorbate peroxidase in the antioxidant protection. *Genetics and Molecular Biology*.

[106] Shao H. B., Chu L. Y., Lu Z. H., Kang C. M. (2008). Primary antioxidant free radical scavenging and redox signaling pathways in higher plant cells. *International Journal of Biological Sciences*.

[107] Hernández L. E., Sobrino-Plata J., Montero-Palmero M. B. (2015). Contribution of glutathione to the control of cellular redox homeostasis under toxic metal and metalloid stress. *Journal of Experimental Botany*.

[108] Jozefczak M., Remans T., Vangronsveld J., Cuypers A. (2012). Glutathione is a key player in metal-induced oxidative stress defenses. *International Journal of Molecular Sciences*.

[109] He J., Li H., Ma C. (2015). Overexpression of bacterial *γ*-glutamylcysteine synthetase mediates changes in cadmium influx, allocation and detoxification in poplar. *New Phytologist*.

[110] Seth C. S., Remans T., Keunen E. (2012). Phytoextraction of toxic metals: a central role for glutathione. *Plant, Cell & Environment*.

[111] Karuppanapandian T., Moon J.-C., Kim C., Manoharan K., Kim W. (2011). Reactive oxygen species in plants: their generation, signal transduction, and scavenging mechanisms. *Australian Journal of Crop Science*.

[112] Yamane K., Mitsuya S., Kawasaki M., Taniguchi M., Miyake H. (2009). Antioxidant capacity and damages caused by salinity stress in apical and basal regions of rice leaf. *Plant Production Science*.

[113] Jaleel C. A., Riadh K., Gopi R. (2009). Antioxidant defense responses: physiological plasticity in higher plants under abiotic constraints. *Acta Physiologiae Plantarum*.

[114] Sofo A., Scopa A., Nuzzaci M., Vitti A. (2015). Ascorbate peroxidase and catalase activities and their genetic regulation in plants subjected to drought and salinity stresses. *International Journal of Molecular Sciences*.

[115] Birben E., Sahiner U. M., Sackesen C., Erzurum S., Kalayci O. (2012). Oxidative stress and antioxidant defense. *World Allergy Organization Journal*.

[116] Trivedi D. K., Gill S. S., Yadav S., Tuteja N. (2013). Genome-wide analysis of glutathione reductase (GR) genes from rice and Arabidopsis. *Plant Signaling and Behavior*.

[117] Potters G., De Gara L., Asard H., Horemans N. (2002). Ascorbate and glutathione: guardians of the cell cycle, partners in crime?. *Plant Physiology and Biochemistry*.

[118] Zitka O., Skalickova S., Gumulec J. (2012). Redox status expressed as GSH:GSSG ratio as a marker for oxidative stress in paediatric tumour patients. *Oncology Letters*.

[119] Li J., Jin H. (2007). Regulation of brassinosteroid signaling. *Trends in Plant Science*.

[120] Harshavardhan V. T., Wu T. M., Hong C. Y., Hossain M., Mostofa M., Diaz-Vivancos P., Burritt D., Fujita M., Tran L. S. (2017). Glutathione reductase and abiotic stress tolerance in plants. *Glutathione in Plant Growth, Development, and Stress Tolerance*.

[121] Cao F., Fu M., Wang R., Diaz-Vivancos P., Hossain M. A., Hossain M., Mostofa M., Diaz-Vivancos P., Burritt D., Fujita M., Tran L. S. (2017). Exogenous glutathione-mediated abiotic stress tolerance in plants. *lutathione in Plant Growth, Development, and Stress Tolerance*.

[122] Ding S., Lei M., Lu Q. (2012). Enhanced sensitivity and characterization of photosystem II in transgenic tobacco plants with decreased chloroplast glutathione reductase under chilling stress. *Biochimica et Biophysica Acta (BBA)—Bioenergetics*.

[123] Reddy A. R., Raghavendra A. S., Madhava Rao K. V., Raghavendra A. S., Reddy K. J. (2006). Photooxidative stress. *Physiology and Molecular Biology of Stress Tolerance in Plants*.

[124] Balakhnina T., Borkowska A. (2013). Effects of silicon on plant resistance to environmental stresses: a review. *International Agrophysics*.

[125] Mourato M., Reis R., Martins L. L., Montanaro G., Dichio B. (2012). Haracterization of plant antioxidative system in response to abiotic stresses: a focus on heavy metal toxicity. *Advances in Selected Plant Physiology Aspects*.

[126] Mahanty S., Kaul T., Pandey P. (2012). Biochemical and molecular analyses of copper-zinc superoxide dismutase from a C 4 plant Pennisetum glaucum reveals an adaptive role in response to oxidative stress. *Gene*.

[127] Myouga F., Hosoda C., Umezawa T. (2008). A heterocomplex of iron superoxide dismutases defends chloroplast nucleoids against oxidative stress and is essential for chloroplast development in arabidopsis. *The Plant Cell*.

[128] Gupta D. K., Palma J. M., Corpas F. J. (2016). *Redox State as a Central Regulator of Plant-Cell Stress Responses*.

[129] Corpas F. J., Palma J. M., Sandalio L. M., Valderrama R., Barroso J. B., del Río L. A. (2008). Peroxisomal xanthine oxidoreductase: characterization of the enzyme from pea (Pisum sativum L.) leaves. *Journal of Plant Physiology*.

[130] Vellosillo T., Vicente J., Kulasekaran S., Hamberg M., Castresana C. (2010). Emerging complexity in reactive oxygen species production and signaling during the response of plants to pathogens. *Plant Physiology*.

[131] Nimse S. B., Pal D. (2015). Free radicals, natural antioxidants, and their reaction mechanisms. *RSC Advances*.

[132] Khelifa S., Hamadi M. M., Rejeb H., Belbahri L., Souayeh N. (2011). Relation between catalase activity ,salt stress and urban environment in *citrus aurantium* L. *Journal of Horticulture and Forestry*.

